# Determination of *ctxAB* expression in *Vibrio cholerae *Classical and El Tor strains using Real-Time PCR

**Published:** 2013

**Authors:** Seyed Mahmoud Amin Marashi, Ramazan Rajabnia, Abbas Ali Imani Fooladi, Zohreh Hojati, Sharareh Moghim, Bahram Nasr Esfahani

**Affiliations:** 1 *Cellular and Molecular Biology Research Center, (CMBRC), Babol University of Medical Sciences, Babol, Iran.*; 2 *Department of Microbiology and Immunology, Babol University of Medical Sciences, Babol, Iran.*; 3 *Infectious Diseases & Tropical Medicine ResearchCenter, Babol University of Medical Sciences, Babol, Iran. *; 4 *Applied Microbiology Research Center, Baqiyatallah University of Medical Sciences, Tehran, Iran.*; 5 *Genetics Division, Biology Department, Faculty of Sciences, University of Isfahan, Isfahan, Iran.*; 6 *Department of Microbiology, School of Medicine, Isfahan University of Medical Sciences, Isfahan, Iran.*

**Keywords:** *Vibrio cholerae*, RT-qPCR, * ctxAB* expression

## Abstract

Cholera is an infection of the small intestines caused by the bacterium *V. cholerae*. It is a major cause of health threat and also a major cause of death worldwide and especially in developing countries. The major virulence factor produced by *V. cholerae *during infection is the cholera toxin. Total mRNA extraction and reverse transcription was performed for making *ctxAB* cDNA. Relative Real-Time PCR analysis showed unequal enterotoxin production in *V. cholerae* strains. The results showed that, classical strain produces cholera toxin more than El Tor strain.

Cholera is one of the infectious diseases that still happens in developing countries. The 8th pandemic of cholera spreads from Southeast Asia across the Middle East and into Central America and Africa (1, 2). The important pathogenesis factor in *Vibrio cholerae* is a potent enterotoxin, cholera toxin, which causes the severe diarrhea of cholera (3, 4). The cholera toxin is produced by *V. cholerae* and CTXΦ phage corporation. Control of enterotoxin gene expression seems to be complex, so that environmental factors are very important in its expression (5, 6). The environmental signals affect *TcpPH* gene and cause its activation and finally affect *ToxT* gene. The ToxT protein is the most important agent for ctxAB toxin expression, because ToxT protein attaches to toxbox region at upstream of *ctxAB* gene and induces ctxAB toxin expression (7, 8) (Fig 1). Beside, other signaling systems such as *ToxR*, *RS*_1_, *AphAB* and quorum sensing have positive or negative effects on ToxT protein (9, 10). Moreover, H-NS protein has negative effect on *TcpPH* and *ToxT* genes that finally would decrease the ctxAB toxin production (11). The RS_1_ region contains *rstA*, *rstB* and *rstC* fragments which have positive effect on *ToxR* gene and therefore increase ctxAB toxin production (12). Bakhshi *et al.* reported several *V. cholerae* attacks in Southwest and Southeast of Iran between 2005-2009 (13, 14). As the level of production of a protein is somehow related to its mRNA quantity, we therefore aimed to determine *V. cholerae* strains that can produce more ctxAB toxin.

**Fig 1:**
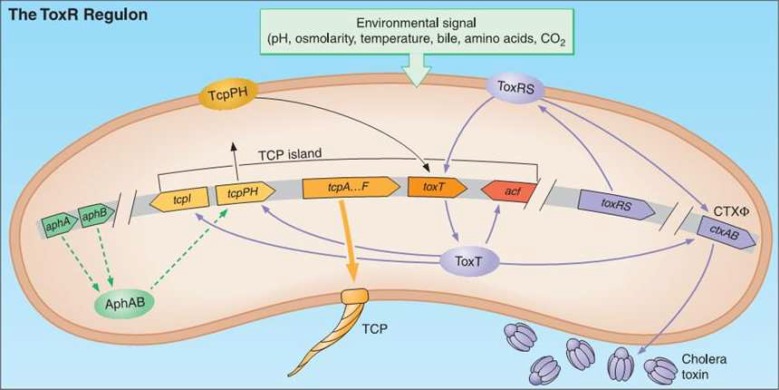
Diagram of the *vibrio cholerae* *ToxR* regulon and *ctxAB* expression, with permission from ASM

## Material and Method

**Bacterial strains and growth conditions**

We used two standard strains named *V.cholerae* O_1 _Classic ATCC 14035 & *V. cholerae* O_1 _El Tor N16961. The isolates were confirmed by biochemical and immunological tests. Serotyping was performed using monoclonal O_1_ antiserum and mono-specific Inaba and Ogawa antisera (Pasteur institute, Paris, France). All selected strains were cultured according to the AKI-SW method and standard growth curve were drawn (15).

**Isolation of RNA and RT-PCR**

Approximately, 2×10^8^ cfu/ml from each strain, was used for total RNA extraction. Total RNA was isolated from the strains isolated randomly from each *V. cholerae *grown in AKI medium using the RNeasy® Protect Bacteria Mini Kit (Qiagen Inc, GMBH, Germany) and the integrity and purity was checked. Equivalent concentrations of total RNA from each strain were selected as template for RT-PCR. cDNA synthesis and PCR amplification were performed using QuantiTec Reverse Transcription Qiagen kit (Qiagen Inc, GMBH, Germany). RT-PCR was performed in the presence of random primer at 42°C for 10 min. After cDNA synthesis, the *ctxAB* and *recA *genes were PCR amplified for checking. PCR amplification was performed for 35 cycles as follows: initial denaturation at 94°C for 5 min, then denaturation at 94°C for 30 sec, annealing at 60°C for 30 sec, extension at 72°C for 30 sec. At the end of the 35th cycle, reaction mixtures were left at 72°C for another 3 min. Five microliters of each reaction mixture was loaded on a 1% agarose gel and subjected to electrophoresis to confirm that the unique amplified fragment correspond to the expected *ctxAB* gene fragment and *recA* as housekeeping gene (16). 

**Real-Time PCR**

Prepared cDNA was quantified using SYBR green I dye. Four primers were designed by AlleleID 6 software, 5’-CAGTCAGGTGGTCT-TATGC-3’ (*ctxAB*-F) and 5’-ATCGTGCCTAAC-AAATCCC-3’ (*ctxAB*-R) for gene of interest and 5’ –ATTGAAGGCGAAATGGGCGATAG- 3’ (recA-F) and 5’ –TACACATACAGTTGGATTGCTTGAGG- 3’ (recA-R) for housekeeping gene. Those primers were specific to *ctxAB *and *recA* and amplified a 115 & 106 bp respectively. SYBR green Real-Time PCR assay was performed with a 20 µl PCR mixture volume containing 2x QuantiTec SYBR Green PCR Master Mix (Qiagen Inc, GMBH, Germany), 0.25 µM specific primer sets, and 2 µl of cDNA sample. Amplification of the primers, data acquisition, and relative analysis were carried out in Chromo4 BioRad Real-Time PCR. PCR reactions were performed as followings: one cycle of 95 °C for 5 min, then 40 cycles of 95 °C for 15 sec, 60 °C for 30 sec. Following the amplification, melting temperature analysis of the PCR products was performed to determine the specificity of the PCR. The standard curve was established by using genomic DNA for each studied gene to confirm that the primers amplified at the same rate and to validate the experiment (55-95°C with warming of 0.2°C per sec). Reverse transcription and PCR positive controls (RNA and DNA, respectively) and negative controls (distilled water) were included in each run. The Real-Time PCR reaction was performed twice assayed in triplicate. Classical *V. cholerae* O_1_ ATCC 14035 was used as a standard control.

## Results

**Fig 2:**
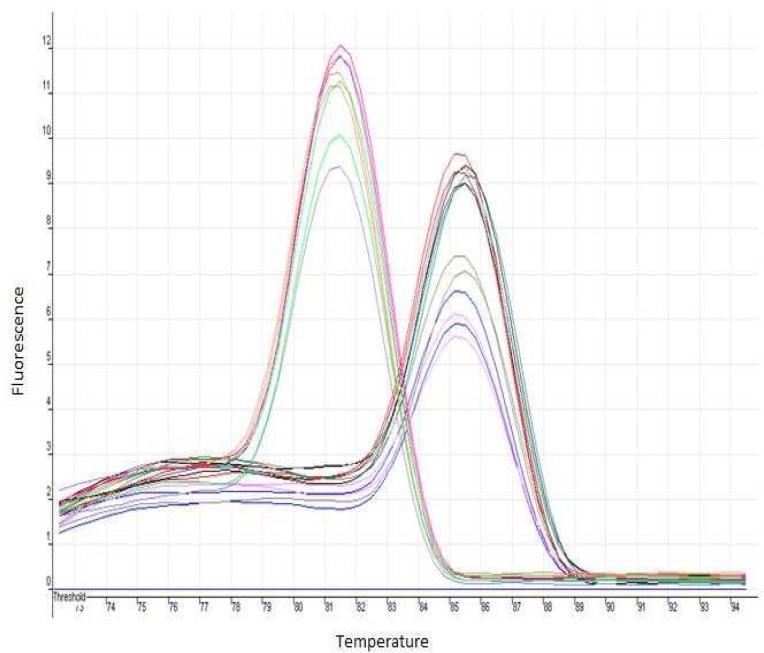
Melting curves of *ctxAB* and *recA* genes

**Fig 3:**
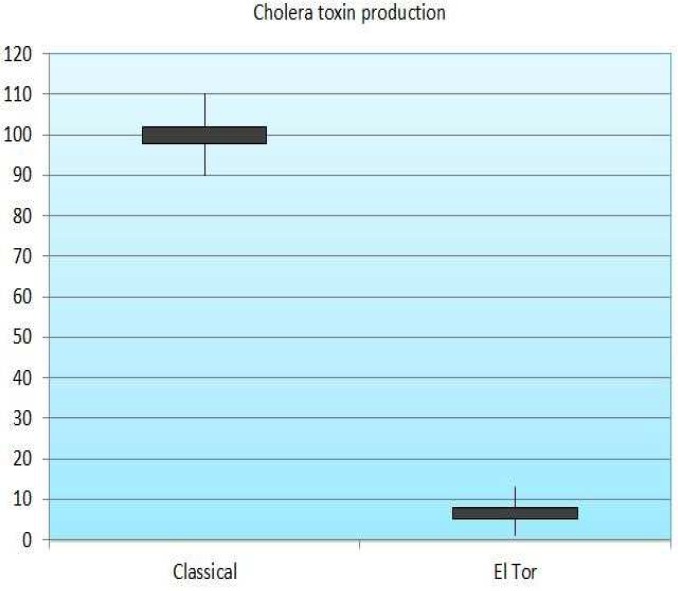
Level of cholera toxin production in classical and EL Tor strains

The specificity of each primer set for *V. cholerae *was tested by PCR with genomic DNA extracted via boiling. Only one size of amplicon was obtained by PCR reaction for *ctxAB *and* recA *genes when DNA from *V. cholerae* strains was used. The amplicons obtained for each gene were verified by sequencing. The presence of a single PCR product was confirmed by Real-Time PCR for each set of the primers using melting curve analysis that resulted in a single product-specific melting curve (Fig 2). The PCR efficiencies varied between 1.90 and 1.94. The relative expression ratio was calculated for each gene of interest by a mathematical model described by ΔC_T _method. The Cycle Threshold (C_T _) results are showed in table 1. Histogram and samples C_T_ values are indicated in Fig 3.

## Discussion

In our study, the results are derived by using “relative” method and ΔC_T _formula. By considering that ctxAB primers have been carefully designed, the amount of standard deviation results are close to zero, the primer-dimer bands were not seen, because the concentration of participating primers in the reaction had been set up. Also, for more accuracy and sensitivity, the PCR efficiency in Real-Time PCR reactions were calculated and replaced with ratio 2 in computational equations.

**Table 1:** Cycle threshold (C_t_) results for the* V.cholerae* O_1 _Classic ATCC 14035 & *V. cholerae* O_1 _El Tor 62013

Strain	Mean C_t_ recA±SD	Mean C_t_ ctxAB±SD	Mean ΔΔC_t_	Mean Ratio*
Classic	25.58±0.14	24.35±0.39	1.23	1.0
El Tor	22.85±0.13	26.02±0.15	4.35	0.06

*ΔΔC_t_ was calculated as: ΔC_t_ (test) - ΔC_t_ (calibrator).Ratio=efficiency ^-ΔΔCt^.

Dirita *et al. *showed that the level of cholera toxin was higher in the classical strain compared to El Tor strain because of the influence of *toxR* on cholera toxin production in the classical strain (17). In our study, in addition to Dirita *et al. *results, we determined quantitative cholera toxin production between classic and El Tor strains. Both classical and El Tor strains have been shown to express equivalent levels of ToxR. In contrast, the classical strain expresses more ToxT, which has a higher binding affinity to toxbox region, resulting in higher expression of cholera toxin (18).

The comparison of the C_T _of the El Tor strain with classical strain shows that toxin production in El Tor strain is approximately 16-17 times lower than in the classical strain (*P *_Value_<0.05 for each). This result is consistent with other reports because the amount of pathogenicity in classic strain is more than El Tor strains (18). Furthermore, histogram and samples C_T_ results indicated that toxin production in classical strain is higher than El Tor strain (Fig 3). In conclusion, the results of our study suggest that other factors modulate the production of cholera toxin by regulating the CTX cassette, supporting the idea that cholera toxin production in* V. cholerae* classical and El Tor strains is a multi-factorial phenomenon.
